# Perceptions of Tennessee cattle producers regarding the Veterinary Feed Directive

**DOI:** 10.1371/journal.pone.0217773

**Published:** 2019-05-31

**Authors:** John E. Ekakoro, Marc Caldwell, Elizabeth B. Strand, Chika C. Okafor

**Affiliations:** 1 Department of Biomedical and Diagnostic Sciences, College of Veterinary Medicine, the University of Tennessee, Knoxville, Tennessee, United States of America; 2 Department of Large Animal Clinical Sciences, College of Veterinary Medicine, the University of Tennessee, Knoxville, Tennessee, United States of America; University of Illinois, UNITED STATES

## Abstract

Since January 1, 2017, the United States Food and Drug Administration (FDA) has fully implemented the Veterinary Feed Directive (VFD) final rule aimed at facilitating the judicious use of medically important antimicrobials in food-producing animals. The objective of this study was to identify the common perceptions of Tennessee (TN) cattle producers regarding the VFD. We used a combination of focus groups and survey questionnaires to explore TN cattle producers’ perceptions regarding the VFD. Preliminary findings from seven focus groups of 62 producers were used in the development of the questionnaire sent both online and in-print to rest of cattle producers in TN. The beef focus group participants perceived the VFD: to be a top-down policy; to have led to unregulated access to in-feed antimicrobials; a regulation that has limited the producers’ ability to prevent disease and leading to economic losses; to negatively affect small producers; and to be affected by challenges related to prescription writing and disposal of un-used medicines. The dairy focus group participants perceived the VFD as unnecessary and burdensome, to have affected small producers, and introduced additional costs. Among the survey questionnaire respondents, 35 (15.4%) beef producers and 6 (13.6%) dairy producers respectively were not familiar at all with the VFD. Forty-eight (21.1%) beef producers and 11 (25%) dairy producers were slightly familiar with VFD. Gender was significantly associated (P = 0.02) with the beef producers’ belief in the usefulness of the VFD. Similarly, for dairy producers, herd size was significantly associated (P = 0.002) with their perceptions regarding the usefulness of the VFD. The findings of this study could inform future VFD policy review processes. More awareness regarding the VFD and its benefits is needed among both beef and dairy producers in TN.

## Introduction

Antimicrobial resistance (AMR) is a major global public health problem [1, 2] that has triggered global concerns over non-judicious antimicrobial use (AMU) in food animals [3]. The association between AMR and is AMU is complex with multiple confounders such as pathogen-drug interactions, pathogen-host interactions, cross-resistance [4, 5]. However, non-judicious AMU and inadequate antimicrobial stewardship (AMS) are known modifiable factors driving the occurrence of AMR [6]. To prevent potential public health consequences of AMR, many countries have instituted measures to reduce and minimize AMU in food animals [6] and have restricted AMU for growth promotion and disease prevention [7]. In Europe, the primary goal of banning the use of antimicrobial growth promoters was to reduce AMR traits in the microbial flora of food-producing animals [8]. Restrictions on the use of medically important antimicrobials in food-producing animals is a major strategy for addressing AMR [9]. The World Health Organization (WHO) recommends complete restriction of AMU in food animals for growth promotion and for disease prevention, and also recommends reduction in the overall use of medically important antimicrobials in food animals [1]. Additionally, The World Organization for Animal Health (OIE) recommends that any AMU in animals should be guided by the OIE standards on prudent and responsible use [10]. The OIE [11] further states that “responsible and prudent use of antimicrobial agents does not include the use of antimicrobial agents for growth promotion in the absence of risk analysis.”

Antimicrobial use restrictions generally aim at mitigating AMR in humans and animals, are often administered through national-level policy [12]. These restrictions are based on the precautionary principle of public health, because there is currently no quantifiable robust evidence of the public health impacts of AMU in food animals on AMR in human pathogens [6]. Recent studies have shown that indiscriminate AMU for both therapeutic and non-therapeutic purposes in animals leads to the propagation and shedding of substantial amounts of AMR microorganisms [6, 13]. In the U.S, there is little published information on the appropriateness of AMU on the farms [14]. Unlike in the Netherlands [15], where administration of antimicrobials to animals is restricted to veterinarians only (except in specified cases), the lack of food animal veterinarians in some areas of the U.S is a challenge to the veterinary oversight of AMU [16].

Beginning January 1, 2017, the United States Food and Drug Administration (FDA) fully implemented the Veterinary Feed Directive (VFD) final rule and all entities regulated by this rule are expected to comply with its provisions. The VFD is aimed at promoting the judicious use of medically important antimicrobials in animals [17]. The VFD only authorizes the use of medically important antimicrobials in feed and water for therapeutic purposes, under the supervision of a licensed veterinarian. In Tennessee (TN), we found that the VFD, and other factors such as producer’ experience and peer support, and antimicrobial drug attributes drive AMU [18, 19]. A previous review that evaluated evidence on the unintended consequences of AMU restrictions in food animals recommended that more research should be conducted to evaluate, document, and report the unintended consequences of interventions targeting AMR reduction [9]. Since implementation, and prior to this present study, U.S. cattle producers’ experiences with the VFD, to the best of our knowledge, had not been studied. No previous study to our knowledge had comprehensively explored and documented the perceptions of TN cattle producers regarding the VFD. Specifically, the objective of the study was to identify the common perceptions of TN cattle producers regarding the VFD. The findings reported here could inform VFD awareness campaigns and could help in the improvement of the VFD and the development of VFD-related policies.

## Materials and methods

### Study design

A mixed methods design using a combination of focus groups and survey questionnaires was utilized. To develop a robust questionnaire that captured our objective, focus group discussions with cattle producers were first conducted to gather opinions about the VFD. Preliminary findings from the focus group discussions were used in the development of the survey questionnaire that was administered to the remaining population of cattle producers in TN. The University of Tennessee Knoxville, Institutional Review Board for the Protection of Human Subjects in Research reviewed and approved both the qualitative (Protocol number: UTK IRB-17-03702-XP) and the quantitative (Protocol number: UTK IRB-17- 03884-XP) parts of this study. Informed consent was obtained from each producer before participation in the study.

### Qualitative methodology

#### Focus group design, structure, and procedure

In total, seven focus group discussions with 62 cattle producers were conducted. Of the seven focus groups, five involved beef producers and two were dairy producer groups. The five-beef producer focus groups were conducted in East TN, Middle TN, and West TN in June 2017 and had a total of 39 participants. For recruitment of beef producers, the leadership of the Tennessee Cattlemen’s Association (TCA) invited members (via e-mail) with experience in different cattle production systems and from different geographical areas to represent a range of beef producers in TN. Each beef focus group comprised of 5–9 producers and lasted approximately 90 minutes. The two dairy producer focus groups were conducted in Middle TN and East TN in July 2017 and March 2018 respectively. The middle TN dairy focus group (dairy focus group 1) was conducted with dairy producers attending an annual dairy producer meeting while participants in the east TN focus group (dairy focus group 2) were recruited from dairy producers attending a master dairy training meeting. Dairy focus group 1 was held in a local restaurant while the second one was conducted at a county extension center. Prior to the dairy producer meetings, the University of Tennessee Institute of Agriculture extension agents notified and requested eligible producers to participate in our focus group meetings. Each focus group meeting lasted approximately 60 minutes. The first dairy focus group comprised 12 producers (participants) while the second one had 11 participants. In both the beef and dairy focus groups, each participant was given an informed consent form with an overview of the study and a signed consent was obtained before participation at the focus group discussion. Participants could opt out of the focus groups at any time. All invited participants were provided with a meal irrespective of their active participation.

A semi-structured interview guide which was modified after the very first focus group was utilized (see S1 File). The modified interview guide (see S2 File) consisted of 11 open-ended questions. We assigned each participant an identity number for confidentiality and to maintain anonymity. These identity numbers were used throughout the discussion and participants announced these numbers before speaking. All the seven focus groups were moderated by one of the authors (EBS) and all the four authors attended each focus group. Three members of the research team (JE, MC and CO) took hand written notes of key points, provided clarifications to questions, and asked follow-up questions were necessary. Debriefing meetings were held at the end of each focus group meeting and before the next focus group discussion as previously described [20]. In the beef focus groups, data saturation was reached during the fifth focus group discussion. However, for the dairy focus groups, we could not determine if data saturation was reached during the second focus group discussion. Data saturation is a point in qualitative data collection and analysis when no new relevant information is obtained from new participants [21, 22]. For thematic analysis, each focus group discussion was video-recorded and later transcribed verbatim by a professional transcription service provider.

### Quantitative methodology

#### Study design and administration of survey

This survey targeted both beef and dairy cattle producers and was part of the broader survey of drivers of AMU practices among cattle producers in TN. First, a questionnaire was developed and evaluated by two professionals with expertise in AMU to ensure all critical issues were identified and covered. Preliminary results obtained from the five beef focus groups and dairy focus group one were used to develop the questionnaire. The questionnaire captured the producer’s demographics and had five questions on producers’ perceptions regarding the VFD (see S3 File for the VFD survey questions). The captured producer demographic information included age, sex (male versus female), level of education, herd size, whether raised on a livestock farm or not, and number of years in cattle farming. A three and a four-point scale as well as ordinal Likert scales were used to capture participant responses to questions on perceptions regarding the VFD.

For beef participants, the sample size required for this survey was determined to be 377 participants at 95% confidence level, a margin of error of 5%, 50% response distribution, and an assumed TN beef producer population size of 20,000 (since the estimated beef producer population size was not known at the time the study was conducted). The survey targeted all dairy producers in the state (the estimated number of dairy producers in TN as of 2017 was 300) [23]. Different participant recruitment strategies were utilized for the two study groups (beef and dairy) because of differences in the population characteristics of the two groups. The survey questionnaire was made available to participants both in print form and online. Producers who completed the print questionnaire were requested in the informed consent statement not to complete the online survey and vice versa. The online version of the survey was housed in a survey software (Qualtrics software, Provo, UT) and was adapted for computer, tablets, and cell phone responses. Participant responses were de-identified using the anonymize function in Qualtrics such that no personal information was collected. Beef producers were notified about the online survey option during the TCA annual meeting in January 2018. Subsequently, all 2,712 producers on the TCA mailing list received an email invitation to take the survey. Additionally, an anonymous survey link and QR code for the online survey were provided to the TCA vice president for distribution to producers willing to take the survey. Dairy producers were also notified about the online survey option during an annual dairy producer meeting in January 2018. Subsequently, an email invitation to take the survey was sent out to all the 87 dairy producers on the University of Tennessee Animal Science department email list. To further increase the response rate, follow-up email reminders were sent to both beef and dairy online survey non-respondents every two weeks.

The printed questionnaire was distributed to beef producers attending the TCA annual meeting, and producer extension meetings across the state and to dairy producers attending dairy extension meetings such as the master dairy training sessions. Completed printed questionnaires were returned to the investigators. The survey (both the printed and online) remained open from January 26, 2018, through May 11, 2018. Participation in the survey was voluntary. All participants were invited to participate in a $10 gift card raffle taken at the end of the survey. The winners were randomly selected and eligibility to participate in the raffle was not contingent upon survey completion.

### Data analysis

#### Qualitative data analysis

The beef transcripts were analyzed using NVivo qualitative data analysis Software; QSR International Pty Ltd. Version 11, 2017, while the dairy focus group transcripts were analyzed using NVivo Version 12, 2018. Thematic analysis was performed using a recursive six-phase approach (familiarization with the data, generation of initial codes, search for themes, review of themes, definition and naming of themes, and report production) as described previously [24]. For data familiarization, each member of the team (JEE, MC, ES and CCO) read all transcripts. The percent of word similarity between the focus groups was assessed using Jaccard’s coefficient. Two separate master projects (beef and dairy) with the transcripts uploaded were developed by the primary author (JEE) and distributed to the other authors for individual coding. For the beef master project, the initial nodes were identified through consensus at the debriefing meetings held after each focus group and each author was at liberty to use either the already prescribed coding frame in the master project (theoretical/deductive approach) or to create new nodes independent of the prescribed coding frame (the inductive approach) during the thematic analysis. For the dairy master project, an inductive approach was used to develop a coding frame (each author created independent nodes). Upon completion of the individual coding, the primary author (JEE) imported the other team members’ coded data into the master project and examined if the themes from the individual coding were related to the coded extracts and all the data transcripts. The degree of agreement in the data coding among the coders (JEE, MC, EBS and CCO) was determined in NVivo using percent agreement. Results harmonization meetings were held by the research team to define and name or re-name themes. The identified themes were refined to identify sub-themes and to ensure that each theme is meaningful, clear and distinct. For the beef focus groups, a thematic map showing the relationship between major and minor themes was created using MindNode, version 5.2.6, mobile application. The findings from these focus groups were reported in accordance to the consolidated criteria for reporting qualitative studies (see S4 File).

#### Quantitative data analysis

A commercial statistical software (SAS, version 9.4, SAS Institute Inc, Cary, NC) was used to complete both descriptive and inferential analyses. The data (see S5 and S6 Files) was summarized using frequencies and proportions. Stacked bar charts created in Tableau software, version 8.2, Seattle, WA and enhanced using adobe art illustrator were used to visualize responses captured on the Likert scale.

To test for associations between the captured demographic information and the producers’ opinions on the usefulness of the VFD policy, univariable and multivariable analyses were performed using ordinal logistic regression. For the univariable analyses, level of education was reclassified into two categories, < college (high school/vocational) or ≥ college, herd size was reclassified into appropriate categories 0–49, 50–99, and > 100 for beef cattle, and < 150 or ≥ 150 for dairy cattle. Age was reclassified into <30, 30–39, 40–49, 50–59, 60–69, and ≥70 using the quantile classification method. In assessing the producers’ opinions on the usefulness of the VFD, a multivariable ordinal logistic regression model was manually fitted using backwards elimination method and the probability of a cattle producer believing that the VFD was less useful was modeled. Spearman's rank correlation was used to evaluate for correlations between predictor variables. For significantly correlated predictor variables, only one was used in the multivariable model building based on completeness of data or ease in interpretation. In the multivariable model building, predictor variables were dropped if they were either non-significant (P > 0.05) or non-confounders. Potential predictors at a P ≤ 0.20 from the univariable analyses were included in the multivariable model building. Possible effects of confounding were evaluated by comparing a change in parameter estimates with and without the suspected confounding variables. A ≥ 20% change in another parameter estimate upon removal of a predictor variable from the model was considered indicative of confounding [25]. The proportional-odds assumption (for the ordinal logistic regression) was evaluated using The Score Test for the Proportional Odds Assumption and the model fit was assessed using Deviance and Pearson Goodness-of-Fit Statistics.

## Results

### Qualitative results

#### Beef producers’ focus group participant characteristics

Of the 39 beef producers who participated in the five focus group discussions, one was female and 38 were male. Focus groups one and two were conducted at Johnson city (East Tennessee) and Dickson county (middle Tennessee) respectively and each had nine participants. Focus groups three and four were conducted at McNairy county (west Tennessee) and Jefferson county (East Tennessee) respectively with eight participants in each of them. Focus group five was conducted at Athens, McMinn county (East Tennessee) and had five participants. Participants’ perceived ages ranged from late twenties to early seventies and the reported herd size per producer ranged from approximately 20 to 225 cattle. Jaccard’s similarity index showed there was diversity among participants in the different focus groups (Jaccard’s similarity index ranged from 27% to 33%). Percent agreement (in coding) between each pair of coders was >75%.

#### Dairy producers focus group participant characteristics

A total of 23 dairy producers participated in the 2 focus groups. Dairy focus group 1 had one female, and 11 male participants while the second one had 2 females and 9 male participants. The reported milking herd size per producer ranged from approximately 40 to 1100 dairy cattle.

The responses from the 2 focus groups were 31.2% similar (Jaccard’s similarity index = 0.312). This Jaccard’s similarity index provided evidence that there was diversity among participants. Percent agreement (in coding) between each pair of coders was > 80%.

#### Beef producers’ perceptions regarding the VFD

**Fig 1:**
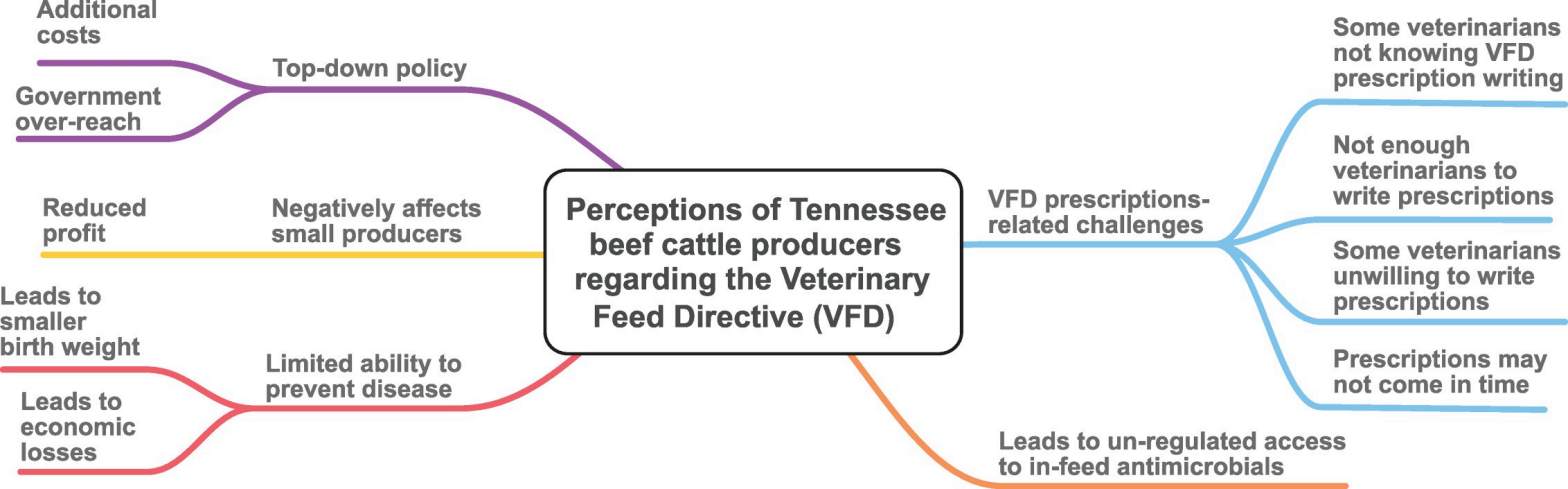
A thematic map showing relationship between major and minor themes for the perceptions of Tennessee beef cattle producers regarding the Veterinary Feed Directive (VFD).

Although a section of participants stated that they were unaffected by the VFD, the VFD was commonly perceived to have negatively impacted production **(Fig 1)**. Broadly, the producers described the VFD: to be a top-down policy; to have led to unregulated access to in-feed antimicrobials; a regulation that has limited the producers’ ability to prevent disease and leading to economic losses; to negatively affect small producers; and to be affected by challenges related to prescription writing and disposal of un-used VFD feed. Below, we give a detailed description and excerpts of the participants’ perceptions about the VFD.

**Top-down policy.** The participants described the VFD as government over-reach that has created additional costs to producers and introduced additional difficulties to producers. Others perceived the VFD as red tape, and a policy that is ineffective. The VFD was also perceived to be a waste of time and money, not only for the producer and the veterinarian, but also for the government.

*…I’m idea on the Veterinary Feed Directive is it did come from the top-down. It was implemented before the education process really even started. And building the plane while you’re flying it doesn’t work*. *It normally results in a crash… [No. 3 focus group 4]*.

*…I’d think they (government) jumped the gun with this VFD deal … [Unidentified participant, focus group 5]*.

The producers also frequently stated that the VFD adds to management by introducing additional labor associated with the work of getting the cattle up to give them an injectable, especially when the cattle may be a long distance away from the handling facilities. Additionally, the VFD was seen to have complicated farm record keeping.

*… [VFD is] Additional hardship and burden on a business already……I think extra cost is all I can see, less profit…. [No. 5, focus group 4]*.

**Unregulated access to in-feed antimicrobials.** Un-regulated access to antimicrobials was mentioned as a likely un-intended consequence (outcome) of the VFD. A section of participants in the west Tennessee focus group mentioned that the VFD would drive some producers to look for alternative sources of in-feed antimicrobials. These alternative sources would mostly be illegal and un-traceable.

*…But you’re gonna cause little things to go kind of illegal to get the job done… [No. 4, focus group 3].…There’s gonna be people that are gonna do things to circumvent law that’s not right… [No. 1, focus group 3]. …That’s when the black market’s gonna [supply in-feed antimicrobials] … [No. 2, focus group 3]*.

**Limited producers’ ability to prevent disease.** The VFD limiting producers’ ability to prevent disease was frequently expressed in all the focus groups. The producers expressed concern that the VFD has disabled disease prevention in their operations and is leading to economic loss and that the VFD is affecting the economic performance of the animals and setting up producers to financial losses. The VFD was commonly mentioned to have negatively affected calf health, led to reduced productivity and negatively affected animal welfare.

*… the VFD has removed an ounce of prevention…They’ve set us up for financial loss… [No. 7, focus group 3]*.

*…It’s [VFD] a loss of money. When we have this in our feed system, our cow[s] were getting treated. …When we have these ingredients [antimicrobials] in our minerals and in our feed, most of the time it helps a lot to keep the pinkeye down, the sore foot down. If they’ve got a sore foot, they’re not going to want to walk to the water trough and to the feed trough. They’re not gaining weight. We’re not making money… [No. 2, focus group 1]*.

The producers also mentioned that, because of the VFD, the lack of access to in-feed antimicrobials for prophylactic purposes would lead to smaller birth weight of calves, and lead to increased culling of calves due to disease.

*…You’d think the public would want to see a healthy calf going to market or a sick calf going to market. That’s what it’s going to be. There’re going to be more and more sick calves slaughtered… [No. 3, focus group 5]*.

**VFD negatively affects small producers.** There was a consensus among all the focus group participants that the VFD has negatively affected the small producers by introducing additional costs of involving a veterinarian and the costs of setting up facilities for handling cattle and therefore, affecting the profit margins of small producers. It was clear from the discussions that small scale beef producers rarely involved veterinarians in their operations.

*… To get the Veterinary Feed Directive, it’s going to require you to have that call. And that small producer–where’s the profit margin at? If you spread that veterinarian client relationship over 100–150 cows, you’re alright. And you have that connection. But if you have nine, that one farm call may have cost you your profit… [No. 4, focus group 5]*.

*…My impression and my opinion is the Feed Directive is particularly impacting negatively the small stocker operation, which is me… If I feed according to script–which we’re probably not going to do anymore–I have to feed 11 pounds per head per day for five days, stop. These calves won’t be eating 11 pounds a week for the first week. [No. 2, focus group 2]*.

*…. A lot of these smaller producers don’t have the facilities to get these animals up. And they might [have] five or ten head of cattle. And if they don’t have that measure in the feed, they don’t have a way of treating them at all. …And their production, if they’ve only got five head of animal[s] and they lose one, that’s 25 percent of their whole herd or 20 percent. That affects their production greatly… [No. 5, focus group 5]*.

**VFD prescription-related challenges.** Some focus group participants commonly expressed concern that some veterinarians did not know how to write VFD prescriptions.

*… And it’s been a nightmare. We get prescriptions that aren’t worth the paper they’re written on. I mean, the vets don’t understand how to write them. And lots of times I have to send an example. They’ll say send me an example of how it should read. I mean, there’s just not been a lot of education on the proper way to write them… [No. 6, focus group 1]*.

*…Even the vets that we deal with didn’t know how to write a VFD. It didn’t have all the items on there that needed to be for us to legally sell the items. If the vets didn’t know how to do it, it’s for sure that the everyday producer didn’t know how it worked. People would come in with the VFD from their vet that wouldn’t even tell what product to give them or what level or quantity to give them. It’s a real struggle, and it still is. We still get those things after months of this that these people don’t know… [No. 5, focus group 5]*.

On the other hand, some producers mentioned that some veterinarians were unwilling to write VFD prescriptions. While others mentioned that in their areas, there are not enough veterinarians to write VFD prescriptions. That even when it is possible to get a VFD prescription, the prescription may be delayed thus limiting their ability to manage disease in their farms. One focus group participant in the McNairy county focus group (West-Tennessee) stated that disposal of un-used in-feed antimicrobials was a challenge because the garbage collectors considered un-used VFD medicines medical waste that is not supposed to be placed in garbage.

*…Some vets won’t write them. They’re just not going to fool with it. It’s just not worth their time …. [No. 6, focus group 1]*.

…I mean, I called the vets. They weren’t around for our program, not in Tennessee. No, sir. I got one, but I never used it. I sent $75.00 to another state and got. A vet in this area would not write one, period… [No. 2, focus group 1].

#### Dairy producers’ perceptions regarding the VFD

The general perceptions from the dairy producer focus groups were that the VFD is an unnecessary and burdensome policy that has affected small producers and introduced additional costs that cannot be passed along to consumers.

*…It’s just more cost. I think it’s $25.00 for the veterinarian–I mean, that $25.00 ain’t [is not] going to make or break nobody. But it’s still $25.00. That’s just something else you gotta deal with. And who gets that? … [No.6, dairy focus group 2]*.

*…There’s no problem with it [VFD] in one sense if I could pass my additional cost along. …You made my cost of production go up. I can’t do a thing about it. I cannot pass that along to the milk processor. I cannot do anything to recoup that cost. I’ve got to bear it all myself… [No.9, dairy focus group 2]*.

The participants mentioned the VFD has limited their access to essential antimicrobial medicines which are necessary for preventive care. This lack of access has led: to increased disease occurrence and deaths especially among calves, reduced productivity, and increased use of injectable antimicrobials.

*…Like on the foot bath for your dairy cows, it’s hard to get the tetracycline now unless you do whatever. That’s our biggest problem because if you don’t keep those warts under control, then you’ve got sore feet. And you’ve got cold cow. That is our biggest problem… [No.8, dairy focus group 2]*.

*… we used to use aureomycin 700. And it was a preventative type thing and a useful thing that we can’t use now. It’s just too much hassle to get it. I couldn’t say that it was that harmful. … [No.1, dairy focus group 1]*.

*…We had to do what we could to get the downtime to try to save our animals*. *We lost some, and we saved some… [No.7, dairy focus group 2]*

However, some producers mentioned that they did not have difficulty accessing these medicines because they have a good veterinarian-client-patient relationship with their veterinarians.

*…Some heifer feeds and other feeds, we go through our vet to get–prescription or whatever you want to call it–even in the beef cattle–mainly Aureomycin that we use in some different feeds. If you have a working relationship with your vet and your vet knows what he’s doing, you don’t have any problems if you’ll do what he says. If you go haphazardly, you’re going to have problems… [No.9, dairy focus group 2]*.

### Quantitative results

#### Beef producers

Out of the required sample size of 377 respondents, a total of 231 (61.3% of the required sample size) beef producers participated in the survey. Of the 231 participants, 103 completed the hard copy survey while 128 completed the online version. Of the 200 participants who responded to the question regarding their gender, 35 were females and 163 were males. Two of these respondents preferred not to report their gender. Complete responses were provided for most questions with the exception of a few cases where the respondents left some questions unanswered. The demographic information of the respondents is presented in **Table 1**.

**Table 1:** Demographics of beef producers on survey of the perceptions of tennessee beef producers regarding the veterinary feed directive.

Variable	Number (%) of respondents
**Gender**	**200**
Female	35 (17.5)
Male	163 (81.5)
Preferred not to report gender	2 (1.0)
**Age group (years)**	**200**
< 30	12 (6.0)
30–39	29 (14.5)
40–49	41 (20.5)
50–59	44 (22.0)
60–69	46 (23.0)
>70	28 (14.0)
**Education level**	**202**
< College	47 (23.3)
≥ College	155 (76.7)
**Years in cattle production**	**202**
< 5	23 (11.4)
6–10	19 (9.4)
11–15	17 (8.4)
16–20	24 (11.9)
21–25	24 (11.9)
26–30	21 (10.4)
>30	74 (36.6)
**Beef cattle operation type**	**230**
Cow-calf production	171 (74.4)
Backgrounding-stocking	9 (3.9)
Seed-stock operation	6 (2.6)
Multiple operation type and others	44 (19.1)
**Herd size**	**202**
1–49	84 (41.6)
50–99	54 (26.7)
100–149	28 (13.9)
150–199	12 (5.9)
200–299	13 (6.4)
300–399	5 (2.5)
400–499	1 (0.5)
500+	5 (2.5)
**Raised on a cattle farm**	**202**
Yes	138 (68.3)
No	64 (31.7)

Of the 228 beef producers who responded to the question on familiarity with the VFD, 35 (15.4%) were not familiar at all, 48 (21.1%) were slightly familiar with VFD, 75 (32.9%) were moderately familiar, 55 (24.1%) were very familiar, and 15 (6.6%) mentioned extremely familiar. A large proportion (36.4%) of respondents were either not at all familiar or slightly familiar with the VFD. Of the 228 beef producers who responded to the question on the usefulness of the VFD, 28 (12.3%) believed the VFD is a very useful policy, 97 (42.5%) believed the VFD is somewhat useful, 32 (14%) took a neutral stand (neither not useful nor beneficial), 27 (11.8%) believed the VFD is not useful. Forty-four producers (19.3%) did not give their opinion on the usefulness of VFD because they were not familiar with the VFD. Of the 227 producers who responded to the question on whether they were aware of the VFD before its implementation, 128 respondents (56.4%) mentioned that they were aware of the VFD before its implementation, eighty-six (37.9%) mentioned they were not aware of VFD before its implementation, while 13 (5.7%) were not sure.

**Fig 2:**
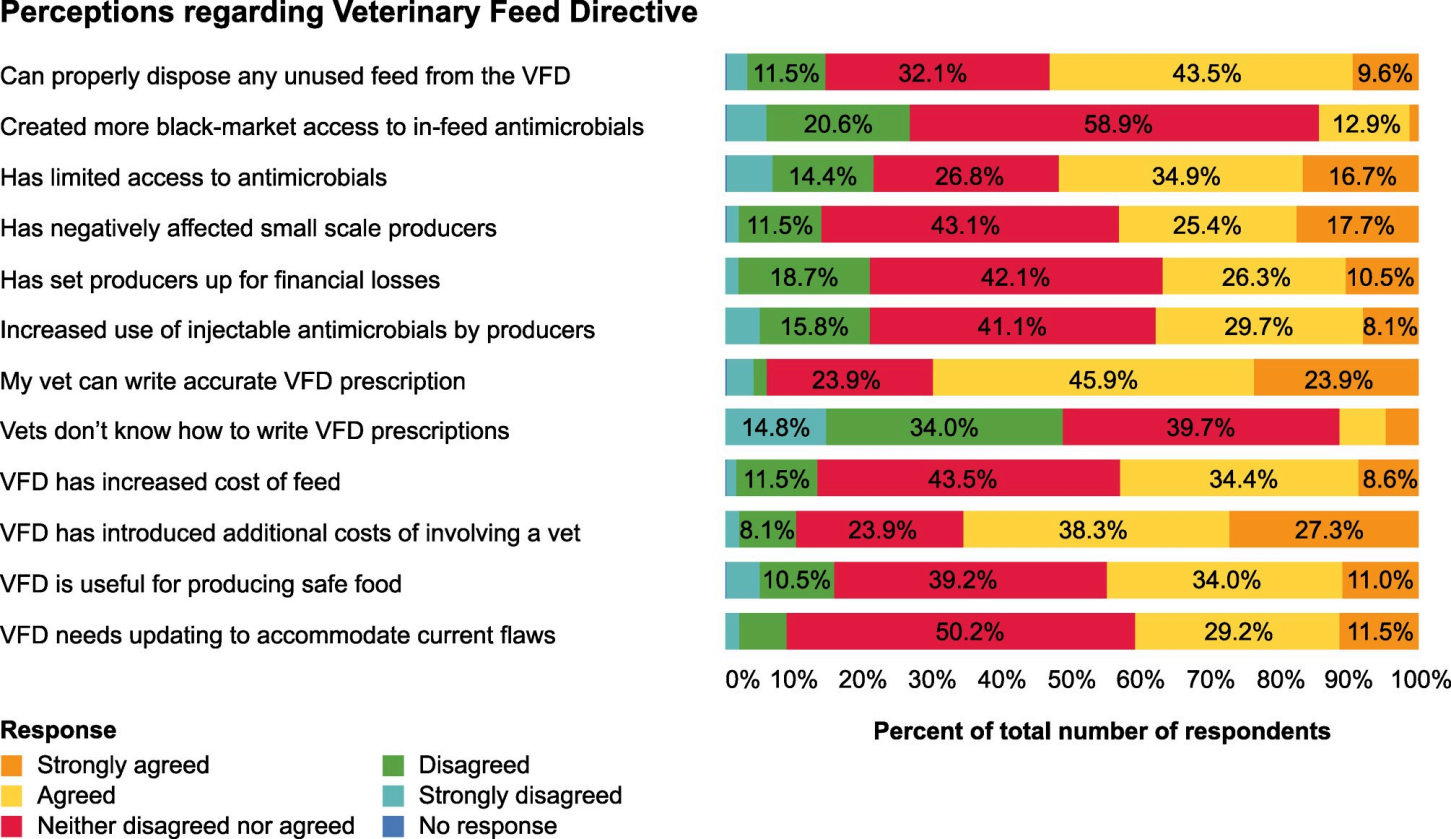
Tennessee beef producers’ perceptions (n = 209) regarding the veterinary feed directive, 2018.

The beef producer responses as to whether the VFD influenced producers to seek veterinary services varied. Out of 223 participants, 45 (20.2%) mentioned that the VFD has caused them to seek veterinarian services more frequently, 137 (61.4%) reported the VFD has not influenced them to seek veterinarian services, 10 (4.5%) reported the VFD has reduced their use of veterinarian services, and 31 (13.9%) did not specify how the VFD influenced their use of veterinary services. More perceptions of the beef producer survey participants regarding the VFD are provided in **Fig 2.**

#### Dairy producers

Overall, the estimated survey response rate for dairy producers was 15%. A total of 45 producers (out of the estimated 300 dairy cattle producers in TN) participated in the dairy section of the survey. Complete responses were provided in most questions except for a few cases where some respondents left some questions unanswered. Of the 45 dairy participants, 40 completed the hard copy survey while only five completed the online version. Of the 39 participants who responded to the question on gender, 31 were males and seven were females. One respondent preferred not to report his/her gender. The demographic information of the survey respondents is presented in **Table 2**.

**Table 2:** Demographics of dairy producers on survey to identify common perceptions of tennessee dairy producers regarding the veterinary feed directive, 2018.

Variable	Number (%) of respondents
**Gender**	**39**
Female	7 (18.)
Male	31 (79.5)
Preferred not to report gender	1 (2.6)
**Age group (years)**	**37**
20–29	2 (5.4)
30–39	6 (16.2)
40–49	8 (21.6)
50–59	13 (35.1)
60–69	8 (21.6)
**Education level**	**37**
High school	16 (43.2)
Vocational	2 (5.4)
College	18 (48.7)
Professional	1 (2.7)
**Years in dairy cattle production**	**38**
< 5	1 (2.6)
6–10	6 (15.8)
16–20	1 (2.6)
21–25	4 (10.5)
26–30	4 (10.5)
> 30	22 (57.9)
**Herd size**	**37**
1–49	2 (5.4)
50–99	8 (21.6)
100–149	7 (18.9)
150–199	5 (13.5)
200–299	7 (18.9)
300–399	3 (8.1)
400–499	1 (2.7)
500+	4 (10.8)
**Raised on a cattle farm**	**39**
Yes	2 (5.1)
No	37 (94.9)

Of the 44 dairy producers who responded to the question on familiarity with the VFD, six (13.6%) were not familiar at all, 11 (25%) were slightly familiar with VFD, 18 (40.9%) were moderately familiar, and nine (20.5%) were very familiar. A substantial proportion (38.6%) of respondents were either not at all familiar or slightly familiar with the VFD. Of the 44 dairy producers who responded to the question on the usefulness of the VFD, one producer (2.3%) believed the VFD is a very useful policy, 10 (22.7%) believed the VFD is somewhat useful, 16 (36.4%) took a neutral stand (neither not useful nor beneficial), nine (20.4%) mentioned that the VFD is not useful. Eight producers (18.2%) did not give their opinion on the usefulness of VFD because they were not familiar with it.

**Fig 3:**
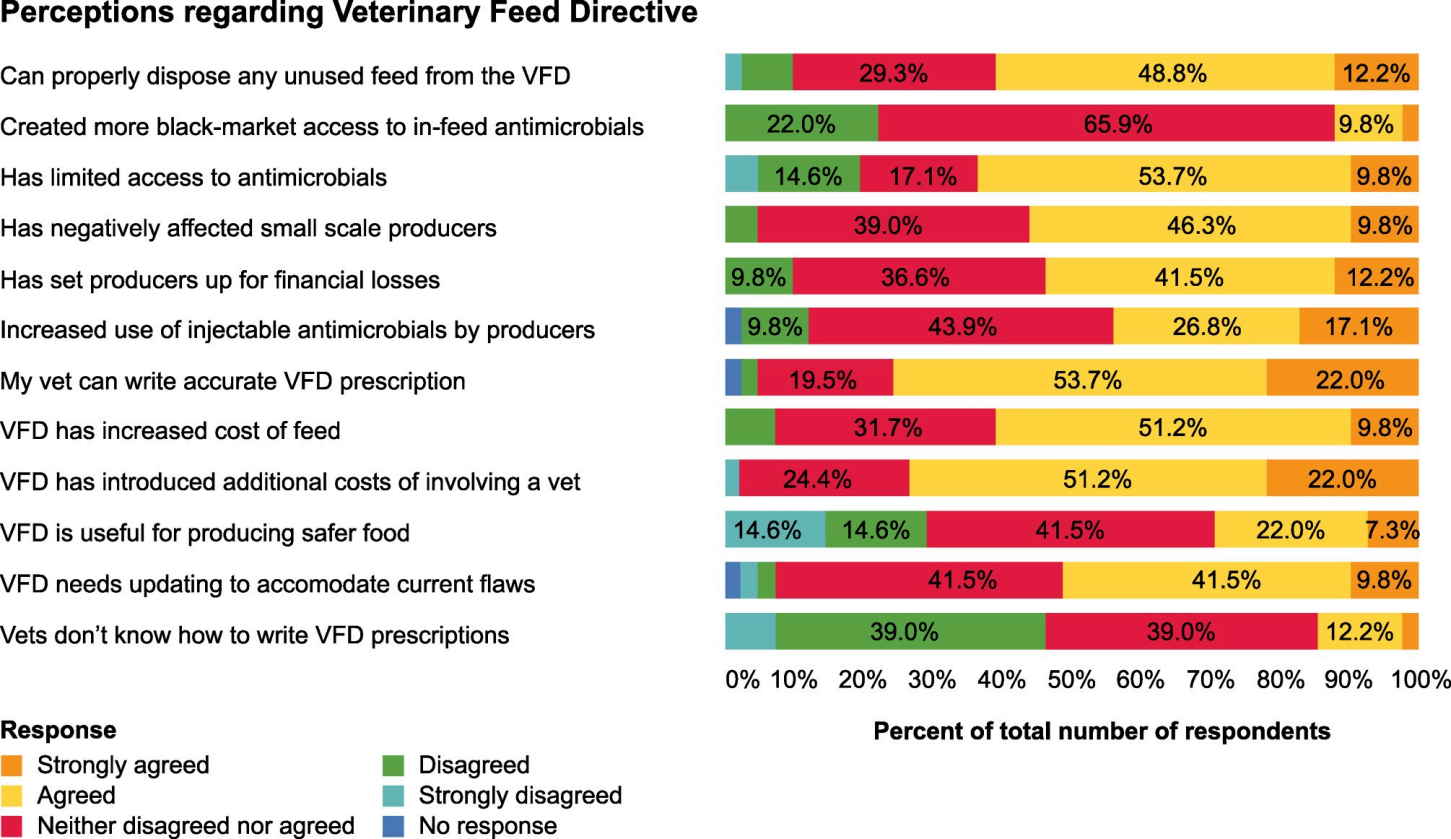
Tennessee dairy producers’ perceptions (n = 41) regarding the veterinary feed directive, 2018.

The dairy producer responses as to whether the VFD influenced producers to seek veterinary services varied. Out of 42 participants, 13 (30.9%) reported that the VFD had caused them to seek veterinarian services more frequently, 23 (54.8%) reported the VFD had not influenced them to seek veterinarian services, four (9.5%) reported the VFD had reduced their use of veterinarian services, two (4.8%) stated that the VFD had not influenced their use of veterinary services in any way. More perceptions of survey participants regarding the VFD are provided in **Fig 3.**

#### Simple associations between demographic variables and producers’ opinions regarding the VFD usefulness

For beef producers, age (P = 0.2), operation type (P = 0.44), number of years in cattle farming (P = 0.19), being raised on a cattle farm (P = 0.77), education level (P = 0.16), and herd size (P = 0.12) were not significantly associated with the producers opinions regarding the usefulness of the VFD policy. However, gender (male vs female; P = 0.02) was significantly associated with the producers opinions regarding the usefulness of the VFD policy. Among the predictors, age of the beef producer and number of years in cattle farming (r = 0.41, P = <0.001) as well as herd size and number of years in cattle farming (r = 0.39, P = <0.001) were significantly correlated. From this univariable analyses, number of years in cattle farming, education level, and gender were considered for inclusion in the multivariable model based on a liberal P-value of approximately ≤ 0.20. For the significantly correlated variables, number of years in cattle farming was chosen for inclusion in the multivariable analyses because of ease of interpretation.

For the dairy producers, age (P = 0.64), number of years in cattle farming (P = 0.22), being raised on a cattle farm (P = 0.39), gender (male vs female; P = 0.96), and education level (P = 0.15) were not significantly associated with the producers opinions regarding the usefulness of the VFD policy. However, herd size (P = 0.002) significantly associated with the producers opinions regarding the usefulness of the VFD policy. Only age of the dairy producer and number of years in cattle farming (r = 0.632, P < 0.001) were significantly correlated. Number of years in cattle farming, education level, and herd size were considered for inclusion in the multivariable model based on a liberal P-value of ≤ 0.20. For the significantly correlated variables, number of years in cattle farming was also chosen for inclusion in the multivariable analyses because of ease of interpretation.

#### Multivariable analyses

In the multivariable analyses, only gender was significantly associated (Odds Ratio for male vs female, 2.47; 95% CI, 1.13 to 5.41; P = 0.02) with beef producers’ belief in the usefulness of the VFD. Implying that compared to females, male beef producers were more likely to perceive the VFD to be a less useful policy. For this beef producer model, the Score Test for the Proportional Odds Assumption (χ^2^(4), DF = 2; P = 0.14) indicated that the proportional-odds assumption was met, and the Deviance (P = 0.14) and Pearson (P = 0.17) Goodness-of-Fit Statistics showed that the model fit the data very well. Similarly, for dairy producers, only herd size remained significantly associated (Odds Ratio for herd size ≥ 150 vs < 150, 24.14; 95% CI, 3.36 to 173.22; P = 0.002) with the perception regarding the usefulness of the VFD following the multivariable analyses. This may imply that dairy producers with herd sizes ≥ 150 were more likely to perceive the VFD to be a less useful policy when compared with those with herd sizes < 150 cattle. For this model, the Score Test for the Proportional Odds Assumption (χ^2^(0.399), DF = 2; P = 0.82) indicated that the proportional-odds assumption was met, and the Deviance (P = 0.69) and Pearson (P = 0.8) Goodness-of-Fit Statistics showed that the model fit the data very well.

## Discussion

The present study identified the perceptions of TN cattle producers regarding the VFD and presents the first published perceptions among cattle producers in TN since the VFD final rule became effective on January 1, 2017. In the present study, the VFD was generally perceived by most producers to have negatively affected them. This finding is similar to the that of a 2015 survey of U.S. beef producers, that was conducted prior to the VFD becoming effective on January 1, 2017 where 70% of the surveyed population expressed a negative attitude towards the VFD [26]. Many participants in the present study were either not familiar or only slightly familiar with the VFD suggesting a need for more producer awareness regarding the VFD. Producers’ negative perceptions regarding the VFD may reflect the challenges and frustrations experienced by the producers since its implementation.

In the present study, TN producers were concerned that the VFD had and would lead to increased occurrence of disease in herds and increased mortalities, has limited their ability to prevent disease, would lead to smaller birth weight of calves, and lead to increased culling of calves due to disease. Although it is reported that the 1986 Swedish ban on the use of antimicrobial growth promoters (AMGP) showed that, with good husbandry practices, profitable animal production can be achieved without the use of AMGP [27], the ban on the use of AMGPs in many countries created the need for alternatives to AMGP (e.g. zinc oxide) [28]. The increased use of high dietary zinc oxide for growth promotion following the AMGP ban has been shown to unintendedly promote AMR [29, 30]. Possibly, as a result of the VFD implementation, U.S cattle producers may end up seeking alternatives to AMGP, which too, could lead to unintended negative effects. A previous review study provided evidence from mostly Europe showing that the unintended consequences from national-level restrictions on AMU on food-producing animals was temporary and minor [9]. Tennessee producers’ concerns regarding the VFD may be justified and warrant more research in other US states. A nationwide evaluation of these perceptions may be useful. Although the intended consequence of the VFD is promoting the judicious use of antimicrobials, its potential negative effects on animal health, welfare, and production could definitely be unintended.

According to the FDA [31], disposal of VFD feed that is no longer needed or is left over should be in a manner that is in accordance with state or local requirements for medicated feeds. In the present study, a beef focus group participant mentioned that disposal of un-used in-feed antimicrobials had become a challenge because the garbage collectors considered un-used VFD medicines to be medical waste that is not supposed to be placed in regular garbage. Similarly, although more than half of the survey questionnaire respondents (both beef and dairy) either agreed or strongly agreed that they were aware of how to properly dispose any un-used VFD feed, some respondents (14.5% beef and 9% dairy) either strongly disagreed or disagreed. These findings suggest that (1) for many TN cattle producers, disposal of un-used VFD feed is problematic, (2) there is a need for more awareness among producers of the FDA guidance on disposal of un-used or expired VFD feed. To ensure proper disposal, veterinarians and beef/dairy extension agents should conduct routine producer awareness regarding the Tennessee requirements (or local area requirements) for disposal of medicated feeds.

In the present study, the producers mentioned that the VFD’s limiting of access to in-feed antimicrobials has affected the economic performance of their herds and would lead to smaller birth weight of calves. Although this concern warrants more research in the U.S. context, a focus on good management practices (including disease prevention) and correct AMU is necessary [27]. It has been suggested that growth response to in-feed antimicrobials is small in optimized production systems [32]. Additionally, changes in antimicrobial consumption following the implementation of policies to discontinue AMU for growth promotion in Denmark did not have a negative impact on swine productivity [33]. Researchers in Europe suggested that unintended consequences, such as illegal AMU practices among producers, may result from the government’s introduction and enforcement of measures (e.g., regulations and fines for contravening the regulations) that aim to induce behavioral change towards judicious AMU [34]. In the present study, some focus group participants mentioned that the VFD “would” lead to un-regulated access to in-feed antimicrobials through the black market. Also, more than 12% of beef participants and more than 9% of dairy producers either agreed or strongly agreed with the statement “the VFD has created more black-market access to in-feed antimicrobials by producers”. Because black market access is possible if there is public demand [35], the farmers assertion that the VFD has created un-regulated access to in-feed antimicrobials through the black market needs to be studied further across the nation so that appropriate interventions to curtail un-regulated access are designed and instituted.

In the present study 37.8% of the beef producers and 43.9% of dairy producers who completed the survey questionnaire either agreed or strongly agreed that the VFD would lead to increased use of injectable antimicrobials by producers. This perception suggests that there might be a compensatory increase in the use of injectable antimicrobials for therapeutic and prophylactic purposes from the time the VFD became effective. It would be beneficial to further investigate the perceived increase in injectable AMU. Based on experiences from Europe, it is recommended that countries that are considering implementing policies that ban AMU for growth promotion, need: (i) to improve veterinary oversight, (ii) to link antimicrobial surveillance to remedial action on excessive AMU, mandatory AMU reduction targets, and (iii) improvements in animal health to contain compensatory increases in AMU [9]. For Tennessee and the U.S. in general, increased campaigns for improved animal health may be the only feasible option for avoiding any compensatory increase in AMU due to the VFD. This is because in TN and the U.S. in general, there is currently: (1) a shortage of food animal veterinarians in some areas, (2) a lack of data on antimicrobial consumption in cattle farms (which data would be an indicator of the appropriateness of AMU), and (3) an absence of mandatory AMU reduction targets.

In the U.S., gender differences in policy preferences are known to exist and are reported to have increased since the 1970s [36]. According to previous findings by May and others, men were more likely to see government policy as excessive when compared to women [37]. In the present study, male beef producers were more likely to perceive the VFD as a less useful policy when compared to females. Possibly, this finding could be because women are more empathic and nurturing, and thus likely to be more concerned about human and animal welfare [38]. Perhaps, this makes them more accepting of policies that are supposed to protect public and animal health. Also, in the present study, dairy producers with herd sizes ≥ 150 were more likely to perceive the VFD as a less useful policy when compared with those with herd sizes < 150 cattle. A possible explanation for this finding could be that, prior to the VFD, dairy producers with larger herds relied more on in-feed antimicrobials for growth promotion on their farms given the higher burden of caring for large herds. For the dairy producers, the variable gender was not considered for multivariable analyses because of its large p-value from the univariable analysis. Possibly, the smaller number of dairy participants did not provide the power required for detecting a gender difference. It is important to note that, for both the beef and dairy producers, only one predictor remained significantly associated with the producers’ perception of the VFD usefulness in the multivariable modeling. Possibly, the reason for this occurrence could be that some important predictors were not measured in the study.

The strengths for this present study were that: (1) there was diversity of opinions among participants as shown by Jaccard’s similarity index and the survey participant demographics, (2) a mixed methods research design was utilized, (3) both focus group and survey respondents were assured that the data collected was anonymized and participation was voluntary, and (4) the survey questionnaire (both print and online) was self-administered. Additionally, the focus group discussions were moderated by one of the authors (EBS) with a background in the behavioral sciences and wide experience in moderating such meetings. Nevertheless, the focus group and survey participants could have given socially desirable responses, thus introducing bias to our findings. Self-selection bias could also be an issue because participants decided entirely for themselves whether or not they wanted to participate in the study [39]. It is likely that the participants in the present study were more interested in the issue of AMR when compared with non-responders. However, bias, if any, could be very minimal. Participants are likely to have given their true perceptions regarding the VFD.

## Conclusions

The findings of this study could inform future VFD policy review processes. Many cattle producers were either not familiar or slightly familiar with the VFD and perceived it as not useful. Disposal of VFD feed, as required of the VFD rule, could be problematic for many TN producers. More awareness regarding the VFD is needed among both beef and dairy producers in TN. For antimicrobial stewardship purposes, campaigns targeting improved animal health in cattle farms should be stepped up to contain the unintended compensatory increase in injectable AMU due to the VFD. A nationwide survey of the perceptions of cattle producers regarding the VFD should be conducted to inform future policy making and implementation, and VFD educational initiatives and awareness campaigns targeting cattle producers.

## Supporting information

S1 File The first focus group interview guide. (DOCX)

S2 File The modified focus group interview guide. (DOCX)

S3 File The demographic and VFD survey questions. (DOCX)

S4 File Consolidated criteria for reporting qualitative studies (COREQ): 32-item checklist. (DOCX)

S5 File Survey raw data for the beef cattle producers. (XLSX)

S6 File Survey raw data for the dairy cattle producers. (XLSX)
