# Supplementary material for: Perceptions of Tennessee cattle producers regarding the Veterinary Feed Directive
Source: PLoS One. 2019 May 31;14(5):e0217773. doi: 10.1371/journal.pone.0217773 (PMC6544306; doi:10.1371/journal.pone.0217773)
Supplement: S1 File — (DOCX) [file pone.0217773.s001.docx]

**S1 file: The first focus group interview guide**

**General questions**

1. Is your current use of antimicrobial agents in feed additives or in treatment of ill animals and how does the Veterinary Feed Directive (VFD) improve or hinder effective your cattle production?
2. How far is your closest food animal veterinarian and does this proximity affect you in any way in light of the VFD regulations?
3. Who or what influences your decision to start (or continue/discontinue) the use of antimicrobials? Take a piece of paper and jot down the factors (things) that are important to you when deciding to use antimicrobial drugs.
4. There is a proposal by certain groups that antimicrobials that are essential for human use, should not be used in animals, even if they are useful to animals. Is restriction of antimicrobial agents for treatment of ill animals in cattle production feasible in your production practice?
5. What can producers, veterinarians, consumers and regulatory authorities do, in order to make antimicrobial use in cattle better?
6. If you are called to give your advice to the secretary of Health and Human Services on antimicrobial resistance problems in food animals and in humans, what do you think are the contributors to the development of antimicrobial resistance problems in food animals and in humans and what advice would you give the secretary for prevention of this challenge?
7. Have you used or thought about using alternative agents that are not antimicrobial agents in your production and what are those?
8. In your opinion, what specific type of information would you as cattle producers need and like to be receiving about antimicrobial use? What is the best format for receiving this information?
9. In one word, describe the current VFD.
10. Of all the things we have talked about antimicrobial use, what is most important to you?
