# Supplementary material for: Perceptions of Tennessee cattle producers regarding the Veterinary Feed Directive
Source: PLoS One. 2019 May 31;14(5):e0217773. doi: 10.1371/journal.pone.0217773 (PMC6544306; doi:10.1371/journal.pone.0217773)
Supplement: S3 File — (DOCX) [file pone.0217773.s003.docx]

**S3 File. The demographic and VFD survey questions.**

Thank you for participating in this study. Please read the attached informed consent statement before proceeding.

I have read and understood the information in the above informed consent statement. Please choose the option below that best represents your consent.

- I agree to participate in the survey and have the chance to win $10 Wal-Mart gift card (If checked, take the survey and then provide your information in the attached form for the raffle drawing).
- I do not agree to participate in the survey, but I want to participate in the raffle for $10 Wal-Mart gift card (If checked, provide your information in the attached form for the raffle drawing).
- I do not agree to participate in the survey and I do not want to participate in the raffle for $10 Wal-Mart gift card (If checked, you may exit the survey).

**Q1.** Which of the following best describes your primary cattle production?

- Beef production
- Dairy production
- Other (specify) ________________________________________________

**Q2.** Which of the following best describes your beef production system? (check all that apply).

- Cow-calf production
- Backgrounding-stocking
- Feedlot operations
- Seed stock
- Other (specify) ________________________________________________

**Q3.** How familiar are you with the Veterinary Feed Directive (VFD)?

- Not familiar at all
- Slightly familiar
- Moderately familiar
- Very familiar
- Extremely familiar

**Q4.** What is your opinion about the Veterinary Feed Directive (VFD)?

- I am not familiar with VFD
- VFD is not useful
- VFD is neither useful nor beneficial
- VFD is somewhat useful
- VFD is very useful

**Q5.** Were you aware of the Veterinary Feed Directive (VFD) before its implementation?

- Yes
- Not sure
- No

**Q6.** How has the Veterinary Feed Directive (VFD), from the time it became effective, influenced your use of veterinary services?

- VFD has reduced my use of veterinarian services
- VFD has not influenced me to seek veterinarian services
- VFD has caused me to seek veterinarian services more frequently
- Other (specify) ________________________________________________

**Q7.** To what extent do you agree or disagree with the following statements?

|  | Strongly disagree | Disagree | Neither disagree nor agree | Agree | Strongly agree |
| --- | --- | --- | --- | --- | --- |
| The Veterinary Feed Directive (VFD) has limited your access to antibiotics |  |  |  |  |  |
| Most veterinarians do not know how to write VFD prescriptions |  |  |  |  |  |
| VFD needs to be updated to accommodate current flaws in execution |  |  |  |  |  |
| My veterinarian can write an accurate VFD prescription |  |  |  |  |  |

|  | | Strongly disagree | | Disagree | Neither disagree nor agree | Agree | Strongly agree |
| --- | --- | --- | --- | --- | --- | --- | --- |
| VFD has introduced additional costs of involving a veterinarian |  | |  | |  |  |  |
| You are aware of how to properly dispose any unused feed from the VFD |  | |  | |  |  |  |
| The VFD would lead to increased use of injectable antibiotics by producers | |  | |  |  |  |  |
| The VFD has created more black-market access to in feed antibiotics by producers | |  | |  |  |  |  |

|  | Strongly disagree | Disagree | Neither disagree nor agree | Agree | Strongly agree |
| --- | --- | --- | --- | --- | --- |
| The VFD has increased the costs of feed |  |  |  |  |  |
| The VFD has negatively affected small scale producers |  |  |  |  |  |
| The VFD has set cattle producers up for financial loss because it has removed access to preventive in-feed medicines |  |  |  |  |  |
| The VFD is useful for producing safer food |  |  |  |  |  |

**Q8.** Which of the following best describes your number of years in cattle farming?

- < 5 years
- 6 - 10 years
- 11 -15 years
- 16 - 20 years
- 21 - 25 years
- 26 - 30 years
- > 30 years

**Q9.** Were you raised on a livestock farm?

- No
- Yes

**Q10.** Which of the following best describes your gender?

- Male
- Female
- prefer not to answer

**Q11.** Which of the following best describes your education level attained?

- No school
- Elementary
- Junior high
- High school
- General Education Development (GED)
- Vocational
- College
- Professional
- Other (specify) ________________________________________________

**Q12.** Which of the following best describes the number of cattle in your production unit?

- 1 - 49
- 50 - 99
- 100 - 149
- 150 - 199
- 200 - 299
- 300 - 399
- 400 - 499
- 500+

**Q13.** Which of the following best describes your age group?

- 19 years and below
- 20 - 29 years
- 30 - 39 years
- 40 - 49 years
- 50 - 59 years
- 60 -69 years
- 70 -79 years
- 80+ years
