# Supplementary material for: Perceptions of Tennessee cattle producers regarding the Veterinary Feed Directive
Source: PLoS One. 2019 May 31;14(5):e0217773. doi: 10.1371/journal.pone.0217773 (PMC6544306; doi:10.1371/journal.pone.0217773)
Supplement: S4 File — (DOCX) [file pone.0217773.s004.docx]

**S4. Consolidated criteria for reporting qualitative studies (COREQ): 32-item checklist**

| **No** | **Item** | **Guide questions/description** |  | **Where in paper** |
| --- | --- | --- | --- | --- |
| **Domain 1: Research team and reflexivity** | | | | |
| **Personal characteristics** | | | | |
| 1 | Interviewer/facilitator | Which author/s conducted the interview or focus group? | All the authors attended all the focus groups. The third author (EBS) moderated the focus group discussions. | Materials and methods. |
| 2 | Credentials | What were the researchers’ credentials? | 1^st^ author (JEE): BVM, MVM, PgD, PhD Candidate  2^nd^ author (MC): BS, DVM, PhD, DACVIM  3^rd^ author (EBS): BA, MSSW, PhD  4^th^ author (CCO): DVM, MS, PhD, DACVPM (Epi) | N/A |
| 3 | Occupation | What was their occupation at the time of study? | JEE: Graduate Research Assistant/PhD Candidate.  MC: Assistant Professor, Large Animal Clinical Sciences.  EBS: Director Veterinary Social work/ Clinical Associate Professor.  CCO: Assistant Professor, Epidemiology and Food safety. | N/A |
| 4 | Gender | Was the researcher male or female | Male: JEE, MC, CCO  Female: EBS | N/A |
| 5 | Experience and training | What experience did the researcher have? | JEE: Underwent qualitative research methods training while at graduate school and has experience in veterinary clinical practice, teaching senior veterinary students at a veterinary school.  CM: Has extensive experience in food animal veterinary practice.  EBS: Has wide experience in moderating group meetings.  CCO: Has wide experience in epidemiology and food safety. | N/A for JEE, MC and CCO.  The experience of the moderator (EBS) is mentioned in the discussion. |
| **Relationship with participants** | | | | |
| 6 | Relationship established | Was a relationship established prior to study commencement | There was no relationship established prior to study commencement. | N/A |
| 7 | Participant knowledge of the interviewer | What did the participants know about the researcher? e.g. personal goals, reasons for doing the research | Prior to the meetings, the participants knew nothing about the researchers. However, at the beginning of each focus group discussion, participants were informed about the purpose of the study as part of obtaining an informed consent prior to commencing with the discussions. | N/A |
| 8 | Interviewer characteristics | What characteristics were reported about the interviewer/facilitator? E.g. bias, assumptions, reasons and interests in the research topic. | Participants were informed that the moderator was a non-veterinarian with a background in the behavioral sciences (social work). | N/A |
| Domain 2: Study design | | | | |
| Theoretical framework | | | | |
| 9 | Methodological orientation and theory | What methodological orientation was stated to underpin the study? e.g. grounded theory, discourse analysis, ethnography, phenomenology, content analysis | For the beef focus groups, researchers were at liberty to use either inductive or the theoretical/deductive approach to thematic analysis.  For the dairy focus groups, an inductive approach was used. | Materials and methods. |
| Participant selection | | | |  |
| 10 | Sampling | How were participants selected? E.g. purposive, convenience, consecutive, snowball | Participants were purposively selected. | Materials and methods. |
| 11 | Method of approach | How were participants approached? E.g. face-to-face, telephone interview, mail, email | Participant recruitment e-mail was sent to the leadership of the Tennessee Cattlemen’s Association (TCA) who then shared this email with TCA members and then purposively selected the volunteers for this study.  Drs. Liz Eckelkamp and Peter D. Krawczel of the Department of Animal Science at the University of Tennessee and Mr. Stan Butt of the Tennessee Dairy Producers Association helped with the mobilization of participants and organizing the dairy focus groups | Materials and methods.  Acknowledgements section |
| 12 | Sample size | How many participants were in the study? | 39 beef producers and 23 dairy producers | Materials and methods. |
| 13 | Non-participation | How many people refused to participate or dropped out? Reasons? | No participant dropped out of the focus groups. | N/A |
| Setting | | | | |
| 14 | Setting of data collection | Where was the data collected? Home, clinic, workplace? | Data was collected at county extension centers or at local restaurants were the focus groups were held. | Materials and methods. |
| 15 | Presence of non-participants | Was anyone else present besides the participants and researchers? | No | N/A |
| 16 | Description of sample | What are the important characteristics of the sample? e.g. demographic data, date | Perceived age: ranged from late twenties to early seventies. | Results |
| Data collection | | | | |
| 17 | Interview guide | Were questions, prompts, guides provided by the authors? Was it pilot tested? | Yes, the interview guide was provided. There was no specific separate pilot testing done. However, the interview guide was modified based on participant comments after the first focus group. | Materials and methods. |
| 18 | Repeat interviews | Were repeat interviews carried out? If yes, how many? | No repeat interviews were carried out. | N/A |
| 19 | Audio/video recording | Did the researchers use audio or visual recording to collect the data? | Data was video recorded. | Materials and methods |
| 20 | Field notes | Were field notes made during and/or after the interview or focus group? | Yes | Materials and methods |
| 21 | Duration | What was the duration of the interviews or focus groups? | The beef focus groups lasted approximately 90 minutes while the dairy focus groups lasted approximately 60 minutes. | Materials and methods |
| 22 | Data saturation | Was data saturation discussed? | Data saturation was reached at the end of the 5^th^ beef focus group. For dairy focus groups, we could not determine if data saturation was reached during the second focus group discussion. | Materials and methods |
| 23 | Transcripts returned | Were transcripts returned to participants for comment and/ or correction? | No. Participants could not be identified since data was de-identified at collection for protection of human subjects in research. | N/A |
| Domain 3: Analysis and findings | | | | |
| Data analysis | | | | |
| 24 | Number of data coders | How many data coders coded the data | All the four authors coded the data | Materials and methods |
| 25 | Description of the of the coding tree | Did authors provide a description of the coding tree? | The coding is described in the manuscript | Materials and methods |
| 26 | Derivation of themes | Were themes identified in advance or derived from the data? | Themes were not identified in advance. Final themes presented in the manuscript were arrived at after review & harmonization meetings to compare individual data coding. | Materials and methods |
| 27 | Software | What software, if applicable, was used to manage the data? | NVivo qualitative data analysis Software; QSR International Pty Ltd. Version 11, 2017 and Version 12, 2018 were used. | Materials and methods |
| 28 | Participant checking | Did participants provide feedback on the findings? | No. Participants were de-identified, hence could not be traced back. | N/A |
| Reporting | | | | |
| 29 | Quotations presented | Were participant quotations presented to illustrate the themes/findings? Was each quotation identified e.g. participant number | Yes, quotations were presented verbatim (in participants’ own words) to illustrate the themes/findings. Each quotation was identified by participant number. | Results |
| 30 | Data and findings consistent | Was there consistency between the data presented and the findings? | Yes | N/A |
| 31 | Clarity of major themes | Were major themes clearly presented in the findings? | Yes | Results |
| 32 | Clarity of minor themes | Is there a description of diverse cases or discussion of minor themes? | Yes | Results |

**N/A: not applicable**
